# Healthcare collapse in Afghanistan due to political crises, natural catastrophes, and dearth of international aid post-COVID

**DOI:** 10.7189/jogh.13.03003

**Published:** 2023-01-11

**Authors:** Ngoc Phuong Hong Tao, Dang Nguyen, Sayed Mansoor Sediqi, Linh Tran, Nguyen Tien Huy

**Affiliations:** 1 Chelsea High School, Chelsea, Michigan, USA; 2 Department of Biomedical Engineering, University of South Florida, Tampa, Florida, USA; 3 Medicine faculty, Kabul University of Medical Sciences (KUMS), Kabul, Afghanistan; 4 Institute of Fundamental and Applied Sciences, Duy Tan University, Ho Chi Minh City, Vietnam; 5 Faculty of Natural Sciences, Duy Tan University, Da Nang City, Vietnam; 6 School of Tropical Medicine and Global Health, Nagasaki University, Sakamoto, Nagasaki, Japan

During the COVID-19 pandemic, Afghanistan's foreign aid-dependent health sector deteriorated with the withdrawal of United States and North Atlantic Treaty Organization (NATO) forces due to the sudden fall of the Afghan government to the Taliban. Although there have been fewer net war-related casualties recently, many people continue to die due to inaccessibility to basic medical care. Political turmoil, catastrophic natural disasters, and the lack of international support have collectively contributed to the collapse of Afghanistan’s health care systems.

Political unrest during COVID-19 increased the scarcity health care professionals. Brain drain, ie, the migration to another country in the pursuit of a better quality of life, has been a concerning issue in Afghanistan for almost two decades of conflicts. There are only 9.4 health care employees per 10 000 patients in Afghanistan, compared to 22.8 workers as the World Health Organization recommends [1]. The massive migration might be predominantly linked to financial instability and safety insecurity during the war-torn and post-conflict era, alongside COVID-19. Prior to March 2022, thousands of medical employees were not paid up to a seven-month salary [2]. Meanwhile, a survey showed that 68% of Afghanistan health care workers (HCWs) used their own budget to buy personal protective equipment [3]. Although being highly at risk of contracting the disease when delivering care to COVID-19 patients, HCWs lack the support of medical supplies from public health authorities to protect themselves and their patients. This might result in an increasingly alarming infection rate and anxiety about personal safety among HCWs, causing an overall burden for understaffed clinics. Additionally, female education has been prevented since the Taliban takeover. Many female nurses, for instance, fled the country to seek higher education and job opportunities due to political instability, lack of funding for pursuing a bachelor's or master's degree, and limited availability of nursing programs [4].

Three decades of conflict, environmental degradation, and inadequate investment in disaster mitigation techniques have significantly increased the vulnerability of many residents during natural disasters. Earthquakes, drought, and flash floods have been highlighted as the most lethal across the country, and the economic implications of COVID-19 are exacerbating humanitarian needs. The most recent combination of natural disasters, including heavy rain, landslides, and earthquakes on June 21, 2022, in the southeastern provinces of Paktika and Khost, was one of the deadliest. This left the country's health system weak and unable to respond through emergency strategies. At least 1036 people were killed, 6083 injured, and 4500 households were destroyed, affecting 361 634 people in 17 districts of Paktika and Khost provinces [5].

There were a variety of health concerns in affected areas before the earthquake. Malaria is endemic in eastern Afghanistan, notably in Paktika and Khost, and measles outbreaks are ongoing. Natural catastrophes have accelerated the spread of communicable diseases and health concerns such as acute watery diarrhoea, measles, tetanus, malaria, and COVID-19. The rising number of home displacements and inaccessibility of water, sanitation, and hygiene (WASH) facilities have exposed the country to a rise in COVID-19 cases and cholera outbreaks [6].

Relying 80% of its budget on external aid, Afghanistan faced major aid suspension once the Taliban took power. The flow of aid in financial assets was halted or reprogrammed by foreign governments and institutions, leaving more than 20 million Afghanistanis requiring humanitarian assistance to survive. Additionally, foreign donors will be unlikely to provide the humanitarian aid that the United Nations (UN) is requesting because many organizations have already departed from Afghanistan [7]. The ongoing collapse of Afghanistan’s economy will terminate decades of efforts to reform the health care system within the country, jeopardizing the well-being of millions of people. 30 million Afghanistanis received medical treatments financed by the World Bank projects, but once the Taliban took power, international donors withheld subsidization that health facilities relied on [8]. Moreover, Afghanistan anticipates closing more than 90% of medical clinics nationwide in 2022, potentially worsening COVID-19 public health measures and contributing to the increase of future disease outbreaks. The UN opened a pledging conference to safeguard the livelihoods of Afghanistanis, reminding the world of the Afghanistan’s situation as the war in Ukraine captured the global community’s attention; this pledging event gathered US$2.4 billion was granted [9]. Nevertheless, only 13% of the objectives delivered in the 2022 Humanitarian Response Plan was acquired through fundraising, highlighting the need to promote urgent financial assistance to process at least until the end of 2022.

**Figure:**
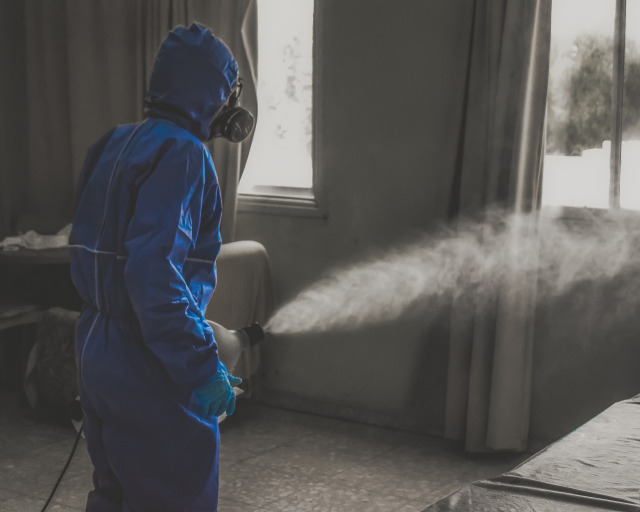
Photo: A health care worker disinfecting a household contaminated with COVID-19. Source: photo by Vetsikas D (2020), available at: https://pixabay.com/photos/covid-19-pandemic-epidemic-5010565/ (free to use under the Pixabay license: https://pixabay.com/service/license/).

With the expansion of the Taliban power, the third wave of COVID-19 compounded on the already crumbled health care system that was neglected by the local authorities. The massive congregation of Afghanistanis desperately seeking refuge at airports, besides the shortage of masks and lack of social distancing, caused a surge in COVID-19-positive cases [10]. Individuals who fled to Kabul lived in crowded settings, with little food, shelter, and hygiene, exacerbating the risk of COVID-19 infection. Many COVID-19 cases were not reported due to poor testing during the Taliban insurgency. Although the country witnessed a 0.52% increase in life expectancy from 2020 (compared to a 0.24% increase in the world’s average) [11,12], WHO predicted that the health status would decline, as health care responses were declining in 2022, and requested global donors to provide comprehensive funding that might enhance medical initiatives in Afghanistan [13]. Previous improvements in health outcomes are likely to be lost, and residents may continue suffering from health care deprivation unless the current political issues, inefficient disaster prevention, and lack of international support are recognized and effectively resolved.
